# Continual learning of multiple cognitive functions with a brain-inspired temporal development mechanism

**DOI:** 10.1093/nsr/nwag066

**Published:** 2026-01-31

**Authors:** Bing Han, Feifei Zhao, Yinqian Sun, Wenxuan Pan, Yi Zeng

**Affiliations:** Brain-inspired Cognitive Intelligence Lab, Institute of Automation, Chinese Academy of Sciences, Beijing 100190, China; University of Chinese Academy of Sciences, Beijing 100049, China; Brain-inspired Cognitive Intelligence Lab, Institute of Automation, Chinese Academy of Sciences, Beijing 100190, China; Brain-inspired Cognitive Intelligence Lab, Institute of Automation, Chinese Academy of Sciences, Beijing 100190, China; Brain-inspired Cognitive Intelligence Lab, Institute of Automation, Chinese Academy of Sciences, Beijing 100190, China; University of Chinese Academy of Sciences, Beijing 100049, China; Brain-inspired Cognitive Intelligence Lab, Institute of Automation, Chinese Academy of Sciences, Beijing 100190, China; State Key Laboratory of Brain Cognition and Brain-inspired Intelligence Technology, Chinese Academy of Sciences, Shanghai 200031, China; University of Chinese Academy of Sciences, Beijing 100049, China; Center for Long-term Artificial Intelligence, Beijing 100190, China

**Keywords:** brain-inspired temporal development, continual learning, evolutionary growth, suppression and pruning, biological synaptic plasticity

## Abstract

Cognitive functions in current artificial intelligence networks are tied to exponential increases in network scale, whereas the human brain can continuously learn hundreds of cognitive functions with remarkably low energy consumption. This advantage partly arises from the brain’s cross-regional temporal development mechanisms, whereby the progressive formation, reorganization and pruning of connections from basic to advanced regions facilitate knowledge transfer and prevent network redundancy. Inspired by these, we propose the continual learning of multiple cognitive functions with a brain-inspired temporal development mechanism, enabling cognitive enhancement from simple to complex tasks in perception-motor-interaction (PMI). The model drives the sequential evolution of long-range inter-module connections to facilitate positive knowledge transfer and employs feedback-guided local inhibition and pruning to eliminate redundancies from prior tasks, thereby reducing energy consumption while preserving acquired knowledge. Experiments on the proposed cross-domain PMI dataset and on general datasets (CIFAR100 and ImageNet) demonstrate that the proposed method achieves continual learning capabilities while reducing network scale, without introducing regularization, replay or freezing strategies, and attains superior accuracy on new tasks compared with direct learning. The proposed method indicates that the brain’s developmental mechanisms provide a valuable reference for exploring biologically plausible, low-energy enhancements of general cognitive abilities.

## Teaser

Inspired by the brain's cross-regional temporal development, the proposed method enables low-energy cross-domain continual learning of multiple cognitive functions via module connection evolution and pruning.

## INTRODUCTION

Artificial intelligence algorithms have achieved remarkable success across various fields, but their enhancement of cognitive functions relies on the massive stacking of parameters. In particular, continual learning systems often struggle to balance memory capacity and energy consumption while adapting to cross-domain tasks [1]. In contrast, the child brain is able to incrementally learn new tasks from different domains while neural connections are being reduced. Neuroscience research has demonstrated that brain structure development follows a specific temporal sequence: different cortical regions mature at different times [2], long-range and local connections exhibit distinct synaptogenic trajectories [3], early-developed regions support later learning [4] and later learning feeds back to regulate early structures [5], ultimately building a lifelong cross-domain cognitive function system.

Extensive progress has been made in multi-task continual learning for artificial neural networks. Methods based on weight regularization [6–8], experience replay [9,10] and weight freezing [11,12] have effectively mitigated catastrophic forgetting in multi-task learning within the same domain. Nevertheless, these approaches often incur substantial computational costs or performance degradation when expanding memory capacity [13]. More critically, the vast majority of existing methods are designed for homogeneous visual classification [14,15] or reinforcement learning tasks [16,17] within a single domain, and thus fail to adapt to continual learning in more realistic cross-domain multi-task scenarios. Therefore, achieving progressive cross-domain multi-task continual learning with high adaptability and low power consumption remains a crucial challenge in the field.

Children gradually acquire cross-domain cognitive functions from simple visual–tactile perception, to bodily motor control, and then to more complex reasoning and decision-making through interactions with the environment [18]. Meanwhile, brain structural development follows a specific temporal sequence: First, intra-regional and inter-regional connectivity exhibit distinct developmental patterns: during childhood, local connectivity undergoes synaptogenesis and reaches a peak at 1–2 years of age, while global transmission efficiency remains low; during adolescence, global connectivity increases and synaptic pruning occurs [19,20]. Second, different brain regions mature at different times: as reflected in the sequential maturation of myelination, dendritic growth and energy metabolism [2]. Primary sensory cortices (e.g. the auditory cortex) reach peak synaptic density and begin pruning early in childhood, whereas higher-order association areas (e.g. the prefrontal cortex) peak much later, around mid-adolescence [21]. Third, bidirectional regulation exists between different brain regions: higher cognitive functions rely on whole-brain dynamic networks formed through the integration of cortical and subcortical structures [4], and higher-order regions, such as the dorsolateral prefrontal cortex, regulate neural activity and structure in primary sensorimotor areas through neurofeedback mechanisms [5,22].

Existing brain development-inspired artificial neural network algorithms primarily focus on fine-grained [23] and structured compression [24], using criteria such as weight magnitude [25], batch-normalization scaling factors [26] and feature similarity metrics [27] to reduce energy consumption. In particular, Brain-inspired spiking neural networks (SNNs), in particular, provide a strong foundational platform for modeling neural mechanisms. This is reflected in a progression of advances ranging from enhanced neuronal dynamics [28,29], to the introduction of hierarchical architectures [30,31], and further to the integration of attention mechanisms and knowledge distillation [32,33], which collectively strengthen spatiotemporal feature extraction and improve overall performance. Current brain-inspired structural optimization algorithms for SNNs can be classified into synaptic plasticity pruning [34], neural activity pruning [35] and pruning-growth fusion algorithms [36]. Although these methods reduce energy consumption, they rely on isolated grow-prune rules and lack mechanisms that draw on large-scale, region-level structural development and long-range developmental sequencing, thereby failing to provide the coordinated developmental dynamics required for efficient knowledge transfer.

To address these limitations, we propose the continual learning of multiple cognitive functions with a brain-inspired temporal development mechanism (TD-MCL). TD-MCL models the inter-regional temporal developmental process of the brain to enable progressive learning of multiple cognitive functions, ranging from perceptual classification to body control and environmental interaction. Specifically, following the developmental principles of brain regions (from primary to advanced) and the hierarchical organization of cognitive tasks (from simple to complex), TD-MCL employs evolutionary algorithms to progressively construct SNN modules and strengthen long-range cross-regional connections. Adaptive learning of interaction patterns between old and new tasks enables previously acquired tasks to facilitate the learning of new tasks. Correspondingly, as learning progresses, feedback from subsequent, more complex tasks guides the transition of local connectivity within earlier task modules from an active to an inhibited state. Numerous local connections associated with early tasks that are not activated in new tasks gradually weaken or disappear, leading to a reduction in overall network size without interrupting continual task learning. We demonstrate that, without introducing continual learning regularization, sample replay or parameter freezing, TD-MCL achieves continual learning in progressively scaled-down networks and enhances the learning performance of new cognitive tasks.

## RESULTS

### Multiple cognitive function dataset

**Figure 1.**
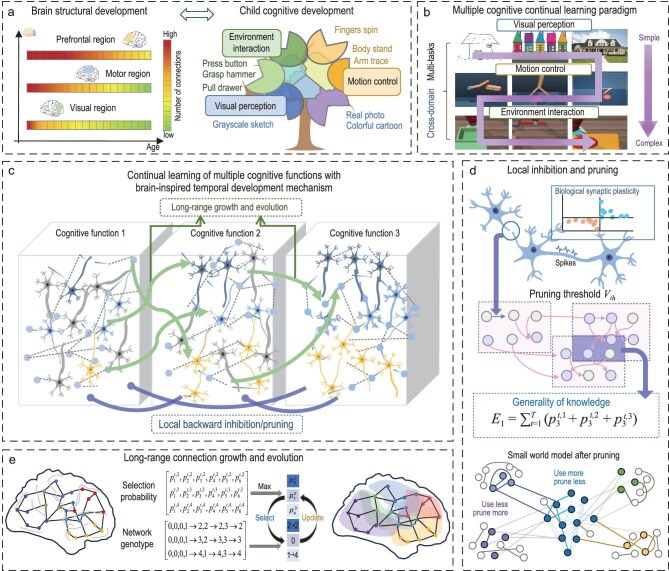
The procedure of the TD-MCL model. (a) Correspondence between the development of cognitive functions and brain structural development in children. (b) Dataset paradigm for continual learning of multiple cognitive functions. (c) General overview of the efficient continual learning algorithm for multiple cognitive functions. (d) Local connection suppression and pruning integrating biological synaptic plasticity and knowledge generalization. (e) Long-range connection growth based on evolutionary algorithms.

The development of children’s cognitive functions exhibits clear temporal characteristics, progressing from basic to advanced in a hierarchical sequence during specific sensitive periods. In the first two months after birth, infants explore the world primarily through sensory input (visual, auditory and tactile) [37]. Between 2 and 10 months of age, strengthened connections between the sensory system and the motor cortex enable infants to perform simple actions such as arm swinging and head turning [38]. After approximately one year, further maturation of the prefrontal and parietal cortices allows infants to actively interact with the environment through grasping and manipulation [39–41]. The development of higher cognitive functions is built upon these primary functions. However, most existing continual learning methods remain limited to a single cognitive domain, failing to reflect cross-domain human cognitive development. To address this limitation, we construct the perception–motor–interaction (PMI) continual learning dataset. Notably, this dataset does not aim to replicate the precise developmental trajectory of infants; rather, it is inspired by their progressive and cross-domain maturation (Fig. 1b).

Specifically, PMI includes nine tasks arranged from simple to complex. In the perception domain, we include three visual recognition tasks—sketch, cartoon and real-image classification [42]—which correspond to the developmental progression of infant visual capabilities from contour processing, to simplified-shape recognition and finally to multi-feature integration [43–45]. The motor domain includes arm-reaching, standing and finger-driven manipulation tasks [46], ordered to reflect the progression from early primary motor cortex development enabling reaching (2–6 months) [47], to cerebellar–motor connectivity supporting balance (2–10 months) [48] and finally to primary–premotor coordination enabling fine finger control (10–12 months) [48]. The interaction domain includes three tasks—button pressing, drawer pulling and hammering [49]—corresponding to infants’ progression in environmental manipulation skills, from spatial targeting, to force modulation and ultimately to tool-based interaction [50,51]. The deep SNN progressively learns these nine tasks during training and is evaluated on all learned tasks during testing.

### Efficient continual learning algorithm for multiple cognitive functions

Cognitive function development parallels brain structural development, as illustrated in Fig. 1a. Inspired by the brain’s multi-scale temporal development, we propose an efficient continual learning algorithm for multiple cognitive functions that incorporates temporally progressive module growth, long-range connectivity growth and evolution, and local connectivity inhibition and pruning, as shown in Fig. 1c. The correspondences between child cognitive functions, neurodevelopmental mechanisms and algorithmic strategies are presented in Table 1.

**Table 1.** Mapping between child cognition, neurodevelopment and TD-MCL modeling.

Child cognitive function	Neurodevelopmental mechanism	Our TD-MCL method
Early fuzzy perception and simple sensorimotor learning	Explosive synaptogenesis: visual $\rightarrow$ auditory $\rightarrow$ motor $\rightarrow$ prefrontal cortex	Progressive modular expansion
Knowledge transfer for new-task learning	Long-range connections gradually strengthened during myelination	Evolution of long-range connections between new and old modules
Ability to learn new tasks despite reduced brain volume	GABA-driven switch from growth to pruning; inactive synapses pruned sequentially; top-down feedback optimizes lower-level circuits	Feedback-guided pruning of learned local connections via Hebbian plasticity and modular reuse

*Temporal progressive module growth.* Neuroscientific research has shown that overall brain synaptogenesis increases rapidly around birth [52,53], but the onset and peak timing of synaptogenesis in different regions occur at different ages [2]. For example, synaptogenesis in the visual cortex increases rapidly between 3 and 12 months of age, whereas synaptogenesis in the prefrontal cortex begins during this period and does not peak until approximately one year of age [54]. Accordingly, the SNN modules in our network are not fixed at the beginning of learning, but instead grow progressively following the task-learning sequence and are connected to form a unified network.

*Long-range connectivity growth and evolution.* During subsequent development, inter-regional long-range communication continued to increase [55,56], facilitating the efficient reuse of knowledge acquired from earlier tasks [57,58]. Accordingly, we employ an online evolutionary algorithm to adaptively enhance long-range connectivity by selecting beneficial modules from previous tasks to support new ones. The evolutionary search space of a new task module (excluding input and output modules) is defined by all candidate connections to previously learned task modules, where each connection is represented as a binary decision variable with two evolutionary states: enabled or disabled. During updating, the probability of selecting connections with higher historical performance and fewer competing alternatives is increased, as shown in Fig. 1e.

*Local connectivity inhibition and pruning.* Low-level cognitive brain regions provide the foundation for higher functions, and the development of higher cognition subsequently optimizes the sparse structure of these regions [5,22]. Guided by developmental timelines, numerous local dendritic spines and synapses that are irrelevant to higher functions are progressively suppressed or pruned [20,52], thereby reducing responses to irrelevant stimuli and enhancing attentional control [59]. For instance, synaptic pruning in the auditory cortex concludes by approximately 12 years of age, whereas pruning in the prefrontal cortex persists until mid-puberty [2]. Inspired by these processes, we introduce a feedback mechanism that performs local connectivity inhibition and pruning on previously learned task modules during the learning of subsequent tasks (Fig. 1d). We quantify connection importance by jointly leveraging two factors: (i) local Hebbian synaptic plasticity, measured by the co-activation strength of pre- and postsynaptic neurons; and (ii) global knowledge generality, captured by the evolutionary selection probability of SNN modules in subsequent tasks. This feedback-guided inhibition and pruning enables the precise identification and retention of globally useful connections while removing redundancies from previously learned tasks.

### Efficient continual learning performance improvement

**Figure 2.**
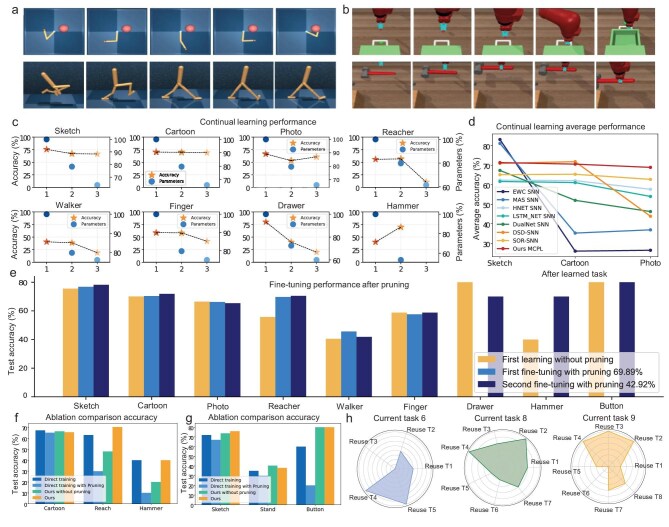
Progressive continual learning performance. (a and b) Examples of successful learning processes for motion control and environmental interaction tasks. (c) Effect of pruning on continual learning performance. (d) Fine-tuning performance of sparse networks after pruning. (e) Comparison with other continual learning methods. (f and g) Performance of ablation experiments. (h) Module reuse determined by long-range connectivity.

Figure 2c presents the corresponding changes in network parameter quantity and performance for the first eight tasks after feedback pruning (with Task 8 undergoing only a single pruning operation guided by the ninth task), without incorporating replay, regularization or freezing operations typically used in continual learning. We set the initial learning rate to 0.1 and adopted a MultiStepLR scheduler (decay factor $\times$0.1), with a total of 80 training epochs.

The results demonstrate that, after feedback-guided inhibitory pruning, the performance of previously learned tasks is largely preserved while the network size for each task decreases exponentially. For instance, the performance of Tasks 1, 2, 3 and 6 remains essentially unchanged, while their parameter counts are reduced to 60.66%, 60.99%, 61.09% and 77.53%, respectively. Notably, Task 4 exhibits a 0.84% performance improvement while retaining 79.81% of its parameters (successful motion control task; Fig. 2a). Similarly, Task 8 achieves a 70.00% success rate despite a substantial 46.89% reduction in redundant network parameters (successful environmental interaction task; Fig. 2b). Although Tasks 4, 5 and 7 experience slight forgetting, they retain effective learning capability. For example, Task 5 shows only a marginal performance decline of 1.93% while retaining 79.73% of its parameters. Overall, the algorithm effectively preserves prior knowledge while enabling new learning as the network becomes increasingly compact and sparse, without relying on conventional continual learning constraints.

To further demonstrate the effectiveness of our approach, we conducted comparative experiments with existing continual learning algorithms. Because our algorithm provides a unified continual learning framework for perception-motion-interaction across cognitive functions, whereas most existing algorithms focus on DNN-based continual learning within a single cognitive domain, we performed fair comparisons in the widely studied visual domain (Fig. 2d). Specifically, we compared our approach with existing SNN continual learning methods (DSD-SNN [11] and SOR-SNN [73]), as well as representative DNN-based continual learning methods—EWC [6], MAS [7], HNET [63], LSTM$\_$NET [64] and DualNet [74]—which we were adapted to the SNN framework.

Our algorithm achieved the highest average accuracy of 68.88% across learned tasks, representing a 6.15% improvement over the second-best performer, SOR-SNN. Although our algorithm did not attain peak performance on the initial simple task, it demonstrated pronounced late-stage acceleration, exhibiting superior capability in learning more complex tasks than competing methods. For example, in the photo classification task, our algorithm achieved an accuracy of 66.00%, outperforming MAS SNN, HNET SNN and DSD-SNN by 12.67%, 20.00% and 3.54%, respectively. Notably, while LSTM$\_$NET SNN, DSD-SNN, EWC SNN and MAS SNN all exhibited substantial performance degradation throughout the learning process, our algorithm maintained the most stable continual learning performance. These experimental results indicate that the proposed algorithm achieves superior performance comparable to existing single-domain continual learning approaches.

To validate the advantages of the sparse architecture and its potential for performance improvement, we conducted a lightweight fine-tuning experiment (20 epochs), as shown in Fig. 2e. Fine-tuning was performed on pruned models of previous tasks when the total pruning rate reached 69.89% (after the fifth task) and 42.92% (after the ninth task). For example, the initial learning accuracy of Task 1 (sketch recognition) was 75.64%. After feedback inhibition across eight subsequent tasks, the pruning rate of the Task 1 module reached 53.79%, yet fine-tuning improved the accuracy to 78.22%. More notably, the initial accuracy of Task 4 (arm tracking) was only 45.28%, but after pruning 49.62% of the connections, the accuracy after the first fine-tuning stage increased markedly to 70.47%. These results demonstrate that our algorithm employs feedback pruning to precisely remove redundant connections while preserving essential synapses from previous tasks, thereby mitigating forgetting and enabling substantial performance gains with minimal fine-tuning.

### General continual learning benchmark improvement

To further verify the task generalization and task-design independence of the proposed brain-inspired temporal development method, we conducted extensive experiments on standard continual learning benchmark datasets, namely Split CIFAR100 and Split ImageNet. Specifically, Split CIFAR100 adopts 10-step (10 classes per step) and 20-step (five classes per step) incremental training settings, while Split ImageNet selects 200 classes from ImageNet to construct the Tiny-ImageNet dataset and divides them into 10 continual learning steps (20 classes per step). All reported results are averaged over multiple independent runs, with the task order randomly shuffled in each run to eliminate potential biases introduced by specific task sequences.

As shown in Table 2, under the 10-step Split CIFAR100 setting, TD-MCL achieves an average accuracy of 87.06%, outperforming the DNN-based method DER++ (84.20%) by 2.86% and the strongest SNN-based method, SCA-SNN (85.61%), by 1.45%, indicating superior stability. Under the longer 20-step task sequence, TD-MCL reaches an accuracy of 89.26%, delivering highly competitive performance relative to DER++ (86.60%) while clearly surpassing several classical methods, such as HAT (85.00%), iCaRL (85.70%) and Mnemonist (86.20%), with average improvements of 2.66% to 4.26%. Moreover, in both settings, TD-MCL outperforms classical regularization- or structure-protection-based approaches, including SI, MAS, OWM and HNET, by more than 10%. TD-MCL also yields standard deviations of only 0.22 and 0.31 in the 10-step and 20-step settings, respectively, demonstrating strong stability and robustness when handling long task sequences.

**Table 2.** Comparative performance of TD-MCL on continual learning for CIFAR100.

	10 steps		20 steps	
Method	Acc (%)	Std (%)	Acc (%)	Std (%)
EWC [6]	61.11	1.43	50.04	4.26
MAS [7]	64.77	0.78	60.40	1.74
TAME [60]	61.06	–	62.39	–
SI [61]	64.81	1.0	61.10	0.83
ERP [62]	–	–	60.08	0.35
OWM [8]	59.90	0.84	65.40	0.07
HNET [63]	63.57	1.03	70.48	0.25
LSTM_NET [64]	66.61	3.77	79.96	0.26
CPG [65]	70.15	3.95	82.60	0.30
PASS [66]	72.40	1.23	76.90	0.77
SGP [67]	76.05	0.43	59.05	0.66
ERK [68]	76.63	0.41	79.61	0.59
OSN [69]	79.08	0.06	63.81	0.06
RDER [70]	80.96	0.34	81.79	0.52
Mnemonicst [71]	82.30	0.30	86.20	0.46
HAT [72]	84.00	0.23	85.00	0.85
iCaRL [9]	84.20	1.04	85.70	0.68
DER++ [10]	84.20	0.47	86.60	0.50
DSD-SNN [11]	77.92	0.29	81.17	0.73
SOR-SNN [73]	80.12	0.25	86.66	0.20
SCA-SNN [12]	85.61	0.24	86.45	0.35
**TD-MCL**	**87.06**	**0.22**	**89.26**	**0.31**

As shown in Table 3, under the more challenging 10-step Split-ImageNet, TD-MCL achieves an average accuracy of 69.1%, improving upon the strongest DNN baseline, RDER (68.8%), by 0.3%, HAT (63.8%) by 5.3% and DER++ (59.7%) by 9.4%. These results demonstrate the superior generalization ability and strong robustness of our method when addressing high-dimensional visual continual learning scenarios. Overall, TD-MCL achieves strong performance on both the multi-domain perception–motor–interaction dataset and standard single-domain benchmarks, demonstrating broad applicability and robust generalization across diverse tasks.

**Table 3.** Comparative performance of TD-MCL on 10-step Tiny-ImageNet continual learning.

Method	Acc (%)	Std (%)
HNET [63]	27.8	0.86
OWM [8]	28.1	0.55
MUC [75]	47.2	0.22
PASS [66]	47.6	0.38
BiC [76]	50.3	0.65
Mnemonicst [71]	52.9	0.66
LwF [77]	55.3	0.35
DER++ [10]	59.7	0.60
HAT [72]	63.8	0.41
SupSup [78]	64.4	0.20
RDER [70]	68.8	0.54
**TD-MCL**	**69.1**	**0.59**

### Cognition processes progress from simple to complex

To demonstrate the superiority of the proposed progressive learning mode from simple to complex, we compare it with direct training and direct pruning optimization methods (i.e. independent learning of individual tasks) applied to the same network, as shown in Fig. 2f and g. The experimental results show that our algorithm achieves the highest performance at the same network size and pruning rate. For example, in the arm motion task, the accuracies of direct training and direct pruning are only 29.9% and 63.02%, whereas our algorithm significantly improves the performance to 70.33% by building on prior cartoon drawing learning. In the button-pressing task, direct training yields only 60% accuracy, which drops sharply to 20% after 40% parameter pruning. By contrast, our algorithm achieves 80% accuracy despite a higher pruning rate of 51.46%, leveraging knowledge from prior stick-figure and standing tasks. These findings highlight the positive role of progressive learning, in which new tasks build upon previously learned ones, in enhancing neural network performance.

This improvement arises from feedback inhibition, which eliminates redundant or irrelevant synaptic connections while selectively preserving shared visual representations through long-range circuits. Accordingly, we further compare the performance with networks that include long-range evolutionary growth but exclude local inhibition (Fig. 2f and g). The ablated networks consistently underperform or fail to match the full TD-MCL algorithm, indicating that the proposed algorithm achieves substantial network compression with only minimal performance loss. In addition, we quantify the evolutionary selection count of long-range connections across nine tasks in Fig. 2h. The results show that Task 6 (finger-related) primarily connects with cognitively related Task 4 (arm) and Task 5 (standing), followed by visually similar tasks such as sketches and cartoons, while avoiding unrelated real photos. Similarly, Task 9 (button pressing) connects mainly with the cognitively related Task 7 (drawer pulling) and Task 8 (hammering), as well as with related arm-movement and color-based tasks.

### Dynamic network connections promote knowledge transfer and reduce energy consumption

**Figure 3.**
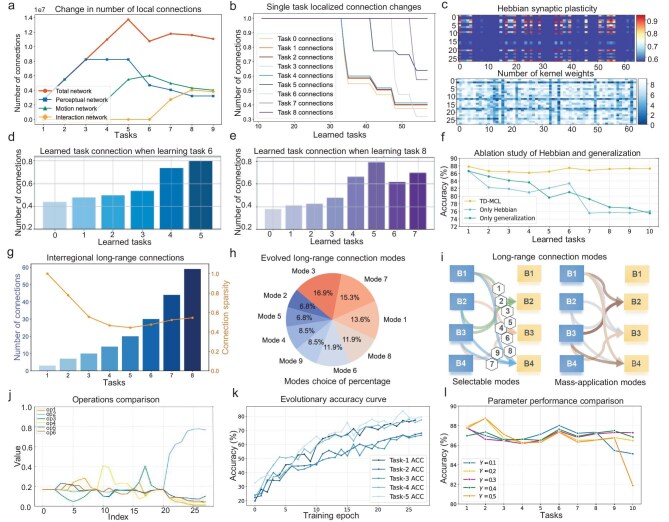
Local and long-range connectivity dynamics. (a) Changes in local connectivity in the overall network and in cognitive-functional networks. (b) Changes in local connectivity in single-task networks. (c) Correlation between biological synaptic plasticity and local connection pruning. (d and e) Number of parameters retained from previous tasks. (f) Pruning ablation study. (g) Long-range connection parameter counts and sparsity. (h and i) Long-range connection mode selection. (j and k) Evolutionary ablation study. (l) Convergence of the evolutionary process.

*Local connectivity dynamics.* To understand the dynamics of feedback pruning and knowledge retention, Fig. 3a illustrates changes in local connections under feedback inhibition and pruning across the overall network and three cognitive functions: perception, motor control and interaction. The results show that the total number of local connections first increases, then decreases and finally stabilizes. This pattern is consistent with biological brain development, in which synaptic connections increase rapidly before approximately two years of age and are subsequently pruned between two and ten years of age, while the capacity for continual learning is maintained [20]. At both the cognitive function level (Fig. 3a, fine line) and the individual task level (Fig. 3b), similar trends are observed. Simple perception modules grow and prune earlier, with higher pruning rates, whereas more complex interaction modules learn later and retain a greater proportion of connections [21]. For example, pruning in the visual module occurs primarily during Tasks 5–7, reducing the final number of parameters to approximately 39.14% of the peak value, whereas pruning in the motor module is concentrated between Tasks 6 and 8, with the post-pruning parameter count representing 66.70% of the peak value. These results indicate that the proposed algorithm’s cross-regional network growth and pruning follow the temporal developmental patterns of the biological brain [2], with feedback-guided pruning removing redundant connections and enhancing attentional processing to improve task performance [79].

From the perspective of forward knowledge transfer, we monitored the post-pruning network size of previous tasks used by the current task (Fig. 3d and e). For example, for motor control Task 6, the pruning rate of the adjacent learned Task 5 is the lowest, whereas that of the initial Task 1 is the highest. This observation suggests that adjacent learned tasks exert the greatest influence on the current task, and that knowledge from earlier tasks progressively transferred through the incremental learning process [80], enabling large-scale pruning of early tasks without significant performance degradation. In addition, we examined the relationship between biologically inspired synaptic plasticity and pruning rates at the convolutional kernel level (Fig. 3c). The results show that the algorithm adaptively prunes a larger proportion of synapses in convolutional kernels exhibiting lower synaptic plasticity.

To validate the effectiveness of the pruning module, we conducted ablation studies in which the local Hebbian plasticity module and the global knowledge generalization module were removed separately in the 10-step Split CIFAR-100 setting (Fig. 3f). The results show that removing the Hebbian plasticity module leads to an 11.25% decrease in average incremental learning accuracy, whereas removing the knowledge generalization module results in an 11.77% decrease. Both declines are substantially larger than those caused by random perturbations. When only local knowledge is available, the initial accuracy is relatively high; however, in the absence of global cross-task information, the average accuracy progressively declines as additional tasks are introduced. In contrast, when only global information is available, the importance of the current task cannot be properly assessed, causing accuracy to fluctuate persistently within a lower range. These results indicate that Hebbian plasticity and knowledge generalization play complementary and indispensable roles in the model, with their respective local and global contributions jointly determining the accuracy and efficiency of the connection pruning process.

*Long-range connectivity dynamics.* Figure 3g shows changes in the number and sparsity of long-range connections during progressive learning. In the progressive learning process, as the number of available task modules for the current task gradually increases, the number of long-range connections gradually increases, consistent with the temporal developmental patterns of steady regional connectivity observed in the biological brain. Notably, the sparsity of long-range connections also increases gradually and stabilizes at approximately 50%. This behaviour indicates that the proposed algorithm selectively connects beneficial modules while avoiding redundant connections, thereby efficiently promoting positive knowledge transfer between tasks and enhancing performance as learning complexity increases.

Moreover, we visualized both the evolution of selection probabilities for different candidate structures (using the third block in Task 2 under the 10-step CIFAR100 setting as an example) and the accuracy trajectories of different tasks during the evolutionary process, as shown in Fig. 3j and k. The results show that the selection probabilities gradually stabilize and increasingly favour superior structures, while the overall model accuracy increases rapidly. These findings further demonstrate that the proposed evolutionary search strategy achieves good convergence and effectiveness. To assess parameter robustness, we further conducted comprehensive ablation studies on the key parameter $\gamma$ (Fig. 3l). Within the range $\gamma \in [0.1,0.5]$, the model’s average incremental learning accuracy consistently remains around 81%. However, an excessively large value of $\gamma =0.5$ induces a certain degree of performance fluctuation due to an overly large update step size of the evolutionary probability, whereas an excessively small value of $\gamma =0.1$ leads to slight performance degradation on later tasks as a result of overly slow probability updates. This indicates that the proposed method exhibits strong stability and robustness with respect to parameter selection.

Figure 3i (left) shows the long-range connection patterns between modules supported by the proposed algorithm. During learning, the evolutionary algorithm exhibits different selection probabilities to these patterns (Fig. 3h and i, right). The most preferred connections integrate the output of the third module from a learned task into the input of the second module in a new task, as well as into the input of the fourth module, thereby facilitating interactions between later-stage modules. In contrast, the second module of the previous task exhibits fewer connections, indicating that the performance-driven evolutionary algorithms are more inclined to select underlying and deeper features for reuse in subsequent tasks. These results show that the long-range connectivity growth promotes knowledge transfer, while local connection inhibition and pruning precisely remove redundant connections from learned tasks, enabling the proposed algorithm to retain acquired knowledge and support continual learning of new tasks under large-scale pruning.

### Computational energy consumption efficiency

To further verify the efficiency of the proposed algorithm, we compare the network energy consumption across methods in terms of the number of connections, floating-point operations per second (FLOPs) and computational energy, as shown in Table 4. The number of connections is reported as the average number of weights activated per task. Computational energy is estimated following the widely used model in [83]. For DNNs, the energy consumption is defined as


(1) \begin{eqnarray*}
E_{\rm SNN}={\rm FLOPS}_{\rm SNN}\times E_{\rm MAC},
\end{eqnarray*}

where $E_{\rm MAC}=4.6$ pJ denotes the energy consumption of a multiply-accumulate (MAC) operation. For SNN, the energy consumption is given by


(2) \begin{eqnarray*}
E_{\rm SNN}={\rm FLOPS}_{\rm SNN}\times E_{\rm AC}\times T,
\end{eqnarray*}

where $E_{\rm AC}=0.9$ pJ represents the energy consumption of an accumulate (AC) operation.

**Table 4.** Energy consumption comparisons for 10-step Split CIFAR100.

	Memory	Number of		Computational
Method	method	connections	FLOPs	energy (pJ)
EWC SNN[6]	Regularization	11.2$\ \times \ 10^6$	1.1$\ \times \ 10^9$	4.0$\ \times \ 10^9$
MAC SNN [7]	Regularization	11.2$\ \times \ 10^6$	1.1$\ \times \ 10^9$	4.0$\ \times \ 10^9$
PODNet DNN [81]	Regularization	11.2$\ \times \ 10^6$	1.1$\ \times \ 10^9$	5.1$\ \times \ 10^9$
iCaRL DNN [9]	Replay	11.2$\ \times \ 10^6$	1.1$\ \times \ 10^9$	5.1$\ \times \ 10^9$
DER++ DNN [10]	Replay	11.2$\ \times \ 10^6$	1.1$\ \times \ 10^9$	5.1$\ \times \ 10^9$
DSD-SNN [11]	expansion	115.8$\ \times \ 10^6$	3.8$\ \times \ 10^9$	13.8$\ \times \ 10^9$
DER DNN [82]	expansion	61.6$\ \times \ 10^6$	6.1$\ \times \ 10^9$	28.1$\ \times \ 10^9$
**TD-MCL**	expansion	8.9$\ \times \ 10^6$	1.0$\ \times \ 10^9$	3.6$\ \times \ 10^9$

The experimental results show that our TD-MCL significantly outperforms existing DNN- and SNN-based continual learning methods in terms of energy efficiency. Specifically, TD-MCL retains only $8.9 \times 10^6$ connections, which is approximately 20% fewer than regularization- and replay-based methods (all $11.2 \times 10^6$), and 6.9$\times$ and 13$\times$ fewer than DER ($61.6 \times 10^6$) and DSD-SNN (115.8$\times 10^6$), respectively. TD-MCL also requires only $1.0 \times 10^9$ FLOPs, slightly lower than other DNN baselines ($1.1 \times 10^9$), and reduced by 6.1$\times$ and 3.8$\times$ compared with DER ($6.1 \times 10^9$) and DSD-SNN ($3.8 \times 10^9$). This advantage primarily arises from two key mechanisms. First, unlike one-shot pruning strategies, our local pruning is repeatedly applied during the learning of subsequent tasks, resulting in pruning rates exceeding 50% for most previously learned task subnetworks. Second, during new task acquisition, the evolutionary algorithm selectively activates only a small number of beneficial network blocks while structurally discarding most connections inherited from earlier tasks, achieving a block-level pruning rate of 57.78%.

In addition, unlike DNN-based methods, the SNN framework replaces multiply–accumulate (MAC) operations with accumulate (AC) operations, which consume less energy under the same FLOP budget. By combining reduced FLOPs with the intrinsic energy efficiency of SNNs, TD-MCL achieves the lowest computational energy among all methods—only $3.6 \times 10^9$ pJ—substantially lower than all DNN and SNN baselines.

In summary, benefiting from local connection sparsity, module-level structural sparsity and the high energy efficiency of SNNs, TD-MCL substantially reduces network energy consumption while maintaining excellent continual learning performance.

## DISCUSSION

Inspired by the brain’s temporal developmental mechanisms, TD-MCL is designed to be forward-looking, enabling continual learning across multiple cognitive functions through brain-inspired structural growth, connection evolution and selective pruning. Biological evidence shows that, as the brain acquires more complex abilities, it actively reorganizes and reuses existing neural structures [4], while long-unused components are pruned to conserve resources [19,20]. Consequently, early simple skills may exhibit mild declines, yet their core functionality is retained, and the system becomes more adaptable for learning higher-level skills. Just as crawling ability diminishes while walking and running abilities improve during human development [84], such selective forgetting constitutes an efficient learning strategy rather than a deficiency. Accordingly, the growth and pruning mechanisms in TD-MCL are not merely engineering choices but computational analogues of developmental neural processes.

Building on these biological principles, TD-MCL overcomes the limitations of traditional single-domain continual learning methods by achieving unified continual learning across perception–motor–interaction tasks. The evolutionary growth of long-range connections enables new tasks to effectively leverage knowledge learned from previous ones, while progressive local pruning reduces network complexity without compromising critical synapses, thereby stabilizing memory retention. Experimental results further validate this design: on the PMI dataset, the accuracy of the visual tasks remains at 66.03% after completing all motor and interaction tasks (compared with 67.10% at the end of the visual stage); on the CIFAR100 20-step benchmark, the average accuracy of the first ten tasks is 88.41% after task 10 and remains 88.81% after all 20 tasks. These findings demonstrate that TD-MCL avoids catastrophic forgetting across heterogeneous tasks, with only marginal performance degradation on earlier tasks.

More importantly, the brain-inspired structural mechanisms of TD-MCL yield strong forward transfer, enabling the model to outperform direct training on complex tasks. As shown in Fig. 2f–g, TD-MCL achieves an accuracy of 80% on the button-pressing task, compared with only 60% under direct training, indicating that selective pruning does not hinder future learning but instead enhances it by reusing beneficial priors and removing redundant or interfering structures.

Compared with existing continual learning methods, TD-MCL does not rely on regularization-based constraints [6,64,85] or replaying samples from previous tasks [86,87]. Unlike structural expansion approaches [11,12], TD-MCL actively prunes redundant connections within previously learned subnetworks during new task acquisition, avoiding parameter freezing while simultaneously reducing computational cost. This provides a new paradigm for incorporating developmental mechanisms of the brain into deep neural networks, enabling efficient and biologically grounded continual learning.

Overall, by introducing brain-inspired temporal developmental principles into artificial neural networks, the structural evolution of the model naturally aligns with known patterns of brain development, yielding more brain-like task adaptivity and energy efficiency while substantially improving continual learning performance without relying on conventional regularization, memory replay or weight-freezing strategies. These results not only validate the effectiveness of developmental brain mechanisms within artificial systems but also provide a novel brain-inspired validation platform for studying developmental processes in neuroscience. From an engineering perspective, brain-inspired dynamic architectures point to promising directions for efficient large-scale models, as exemplified by the mixture-of-experts (MoE) design in DeepSeek. Building on this insight, our future work will integrate interpretable, brain-inspired structural evolution into large-scale models to improve their energy efficiency, adaptability and generalization.

## METHOD

### Adaptive evolution of long-range connectivity

To avoid task interference caused by redundant connections and insufficient knowledge reuse due to missing connections, we do not manually design long-range connections between task modules. Instead, we adaptively learn increasing inter-regional connections via online evolution. Specifically, we evolve connections between new modules in ResNet18 blocks 2–4 and existing modules from prior tasks (block 1 receives the input). For each task *t*, new module *b* selects connections to modules from a prior task *k*, governed by a probability vector $P_{b}^{t,k}$, as follows:


(3) \begin{eqnarray*}
P_{b}^{t,k}=[p_1,p_2,p_3,p_4,p_5,p_6].
\end{eqnarray*}

Here, $p_1,p_2,p_3$ denote probabilities of not connecting to any module from prior task *k*, yielding 50% long-range connection sparsity (which is biologically consistent). Probabilities $p_4,p_5,p_6$ represent connections to the three modules of task *k*. Task *t* adds $3(t-1)$ nodes (each with three evolvable edges ($M=2$ operations: connect or not connect). The connection probability matrix (from task *t* to prior tasks) has a size of $3\times (t-1) \times 6$, enabling efficient knowledge transfer and biological plausibility.

Connection probabilities are updated according to loss performance from the current selections. First, the historical selection count $h_n$ is recorded, and a performance metric $h_l=1-{\rm Normalize}_{\mathrm{0--1}}({\rm loss})$ is defined, where the accumulated epoch loss is min-max normalized to [0,1]. The selection counts and performance differences for each connection are then compared:


(4) \begin{eqnarray*}
d_{h_n}= h_n - {h_n}^T,\quad d_{h_l} = h_l - {h_l}^T,
\end{eqnarray*}

where ‘$T $’ denotes the transpose operation. If a connectivity pattern *i* is selected less frequently but yields better performance than *j*, we define $i \succ j$ and increase its probability; otherwise, its probability is decreased:


(5) \begin{eqnarray*}
dp^{+} = \sum (d_{h_n}< 0 \wedge d_{h_l} > 0) =\mathbf {1}(i \succ j),
\end{eqnarray*}

(6) \begin{eqnarray*}
dp^{-} = \sum (d_{h_n} > 0 \wedge d_{h_l} < 0) =\mathbf {1}(j \succ i).
\end{eqnarray*}

The learning rate $\gamma$ is used to update the connection probabilities, which are subsequently normalized using the softmax function:


(7) \begin{eqnarray*}
p= {\rm Softmax}(p + \gamma \cdot (dp^{+} - dp^{-})).
\end{eqnarray*}

### Inhibition and pruning of local connections

According to the principle of ‘use it or lose it’, we first calculate the Hebbian synaptic plasticity, which is composed of the spiking traces of presynaptic and postsynaptic neurons:


(8) \begin{eqnarray*}
{\rm Trace}_i^{{\rm step}}= \alpha {\rm Trace}_i^{{\rm step}}+ S_i^{{\rm step}},
\end{eqnarray*}

(9) \begin{eqnarray*}
H_{ij}={\rm Normalize}_{0-1}({\rm Trace}_{i}^{{\rm Step}} \cdot {{\rm Trace}_{j}^{{\rm Step}}}^T),
\end{eqnarray*}

where $\alpha$ is the decay coefficient. Indices *i* and *j* denote post-synaptic and pre-synaptic neurons, respectively, while *step* and *Step* represent the spike timing step and the total length of the spike time window.

In addition to local spiking activity, we also consider the global knowledge generalization $E_b^k$ of a module across tasks, defined as the sum of the probabilities that a module from learned task *k* will be reused in subsequent tasks (up to the current task *t*), as given in


(10) \begin{eqnarray*}
E_b^k=P_b^{k+1,k}+P_b^{k+2,k}+\cdots +P_b^{t,k}.
\end{eqnarray*}

The usage probability of evolved modules reflects their knowledge generalizability, and pruning redundant modules improves efficiency by removing non-essential connections.

Furthermore, as brain pruning and inhibitory processes gradually stabilize with age, we combine local synaptic plasticity, global generalizability and the number of training runs to compute synaptic threshold coefficients, constrained to the interval [0,1], as follows:


(11) \begin{eqnarray*}
V_{ij}=\min (1,n/N) (1 - e^{-2(H_{ij}+E_b^k)}).
\end{eqnarray*}

These synaptic threshold coefficients are used to inhibit synapse weights or even prune synapses to varying degrees, improving network task focus and reducing overfitting:


(12) \begin{eqnarray*}
w_{ij}^{\prime }={\rm Sign}(w_{ij}){\rm Relu}(|w_{ij}|-V_{ij}|w_{ij}|_{{4}/{5}}),
\end{eqnarray*}

where $w_{ij}$ and $w_{ij}^{\prime }$ denote the original synaptic weight and the weight after inhibition and pruning, respectively. Here, $|w_{ij}|_{{4}/{5}}$ denotes the 0.8 quantile of the absolute value of synaptic weights.

Further methodological details, including dataset settings, spiking neural networks, progressive module expansion and theoretical analysis, are provided in the online supplementary material.

## Supplementary Material

nwag066_Supplemental_File

## Data Availability

The data used in this study are available from the following databases. The perception cognitive task data [42] are available at https://github.com/robertofranceschi/Domain-adaptation-on-PACS-dataset. The motion cognitive task data from the DeepMind Control Suite environment [46] are available at https://dl.fbaipublicfiles.com/eai-vc/mujoco_vil_datasets/dmc-expert-v1.0.zip. The interaction cognitive task data from the MetaWorld environment [49] are available at https://dl.fbaipublicfiles.com/eai-vc/mujoco_vil_datasets/metaworld-expert-v1.0.zip.
