# Supplementary material for: Continual learning of multiple cognitive functions with a brain-inspired temporal development mechanism
Source: Natl Sci Rev. 2026 Jan 31;13(7):nwag066. doi: 10.1093/nsr/nwag066 (PMC13101990; doi:10.1093/nsr/nwag066)
Supplement: nwag066_Supplemental_File [file nwag066_supplemental_file.pdf]

Supplementary Data for Continual Learning of Multiple Cognitive Functions with Brain-inspired Temporal Development Mechanism

1 Multiple Cognitive Function Dataset Setup

To simulate the continual enhancement of various cognitive abilities in the brain, we have designed a cross-domain perception-motion-interaction multiple cognitive function dataset. In the perception domain, we utilized three visual classification datasets, including sketches (3,929 images), cartoon drawings (3,929 images), and real photographs (1,670 images) [1], with image samples as inputs and recognition accuracy as the performance metric. For the motion domain, we employed the stand and walk tasks of the Walker agent and the Reacher agent task from the DeepMind Control Suite environment[2] in Mujoco. Leveraging the image feature extraction capabilities learned from previous tasks, we also used images as inputs in motion tasks, learning new feature extraction while adaptively reusing the previously acquired basic feature extraction modules for color images. Each task was provided with 47,500 example videos and action output labels for supervised learning during training. During testing, actions were determined based on real-time environmental image inputs, with average return reward as the performance metric. In the interaction domain, building upon the acquired image and motion control capabilities, we used 10,000 example video images and state inputs from the drawer-open, hammer, and button-press tasks in the metaworld environment[3] during training. The outputs were continuous command sequences (real values). During testing, actions were similarly determined based on environmental images and states, with task success rate as the performance metric.

This comprehensive dataset and methodology aim to provide a robust framework for studying and enhancing multiple cognitive functions, facilitating advancements in artificial intelligence and cognitive science research.

2 SNN Architecture Progressive Development

Brain-inspired algorithms have garnered significant attention , among which Spiking Neural Networks (SNNs) provide a suitable foundational architecture. SNNs utilize

brain-inspired spiking neurons as their basic units, transmitting information through discrete 0/1 spikes. As a result, SNNs exhibit high biological plausibility, energy efficiency, and strong adaptability to hardware platforms. In this paper, we employ the Parametric Leaky Integrate-and-Fire (PLIF) neuron [4], with the membrane potential U_i and spike S_i as Eq. 1-2, to construct a ResNet18 network architecture. The network weights are updated using a surrogate gradient algorithm, which enables effective training of the SNN while maintaining its spiking nature and computational efficiency.

$$U_i^{step} = \sigma(\tau) U_i^{step-1} + \sum_{j=1}^{M} P_t^{ij} S_j^{step}, \tag{1}$$

$$S_i^{step} = \begin{cases} 1, & U_i^{step} \geq V_{th} \\ 0, & U_i^{step} < V_{th}. \end{cases} \tag{2}$$

Where τ is a learnable parameter, $\sigma(\cdot)$ denotes the sigmoid function, and V_{th} represents the spike firing threshold.

During the first two years after birth, the infant brain continuously forms new connections to establish a structural foundation for progressively mastering diverse cognitive tasks [5]. Inspired by this developmental mechanism, our algorithm similarly grows network modules to acquire previously unknown knowledge when learning new tasks t. Specifically, we expand the network horizontally at the block level for each convolutional layer, with the number of convolutional channels increasing by 32-64-128-256 across the four blocks of ResNet18 as Eq. 3. New modules are integrated via long-range connections to blocks associated with existing tasks, as detailed in the next section.

$$\{B_1^t, B_2^t, B_3^t, B_4^t\} \rightarrow \begin{cases} B_1^1, B_2^1, B_3^1, B_4^1 \\ \vdots \\ B_1^{t-1}, B_2^{t-1}, B_3^{t-1}, B_4^{t-1}. \end{cases} \tag{3}$$

3 Adaptive Evolution Theoretical Analysis

Regarding complexity, taking the 10-step Split CIFAR-100 setting as an example, since the evolutionary process proceeds incrementally across tasks, the average number of candidate edges per task $E = 40.5$. From this, we derive that the average time complexity of the evolutionary update per epoch throughout the entire training process is:

$$T_{EVO} = O(|E| \cdot M^2), \tag{4}$$

which is negligible compared with the standard backpropagation complexity:

$$T_{BP} = O(\frac{N}{B} \cdot C_{step}), \tag{5}$$

where N is the dataset size, B is the batch size, and C_{step} denotes the computational cost of one forward–backward pass.

Algorithm 1: The long-Range connection evolution algorithm

Input: Dataset D^t_{train} and D^t_{test}.
Output: Prediction class y.
for t *in sequential task* N **do**
  for e *in Epoch* **do**
    $g_e = max(p), loss = 0$
    for b *in Batch* **do**
      $y = SNN(D, g_e)$ with local connection pruning;
      loss+=Backpropagation(SNN);
    end
    $h_l = 1 - Norm(loss)$
    $h_n = h_n[g_e] + 1$
    $d_{h_l} = h_l - h_l^T, d_{h_n} = h_n - h_n^T$
    $dp^+ = \sum((d_{h_n} < 0) \wedge (d_{h_l} > 0)$
    $dp^- = \sum((d_{h_n} > 0) \wedge (d_{h_l} < 0)$
    $p = Softmax(p + \gamma(dp^+ - dp^-))$
  end
end

Regarding convergence, the update of the selection probability for structure can be mathematically interpreted as a rank-based pairwise preference optimization. Let s_i denote the latent quality of operation i. Using the Bradley–Terry model, the probability that i is preferred over j is:

$$P(i \succ j) = \frac{\exp(s_i)}{\exp(s_i) + \exp(s_j)}. \tag{6}$$

The pairwise preference label $y_{i,j}$ is determined by the empirical statistics h_n, h_l. Minimizing the standard pairwise ranking loss:

$$\mathcal{L} = -\sum_{i,j} [y_{ij} \log P(i \succ j) + (1 - y_{ij}) \log P(j \succ i)] , \tag{7}$$

yields the gradient:

$$\Delta s_i \propto \sum_{j \neq i} (y_{i,j} - y_{j,i}) , \tag{8}$$

which, after projecting to the probability simplex, corresponds exactly to our update:

$$p_i \leftarrow p_i + \alpha \sum_{j} [\mathbf{1}(i \succ j) - \mathbf{1}(j \succ i)] . \tag{9}$$

Therefore, our update rule can be viewed as a stochastic approximation to the gradient of the expected architecture reward, using pairwise preference as an unbiased estimator. The complete algorithm flow for long-range connection evolution is as Algorithm 1

References

[1] Zhou YYHTXT Kaiyang. Deep domain-adversarial image generation for domain generalisation. *Proceedings of the AAAI Conference on Artificial Intelligence*, volume 34 (2020) 13025–32.

[2] Tassa DYMAETLYCDdLBDAAMJLAo Yuval. Deepmind control suite. *arXiv preprint arXiv:1801.00690* 2018; .

[3] Yu QDHZJRHKFCLS Tianhe. Meta-world: A benchmark and evaluation for multi-task and meta reinforcement learning. *Conference on Robot Learning* (2020) 1094–1100.

[4] Fang YZCYMTHTTY Wei. Incorporating learnable membrane time constant to enhance learning of spiking neural networks. *Proceedings of the IEEE/CVF International Conference on Computer Vision* (2021) 2661–71.

[5] Sakai J. How synaptic pruning shapes neural wiring during development and, possibly, in disease. *Proc Natl Acad Sci USA* 2020; **117**: 16096–99.
